# Antioxidant and Cytoprotective Properties of Plant Extract from Dry Flowers as Functional Dyes for Cosmetic Products

**DOI:** 10.3390/molecules26092809

**Published:** 2021-05-10

**Authors:** Tomasz Bujak, Martyna Zagórska-Dziok, Aleksandra Ziemlewska, Zofia Nizioł-Łukaszewska, Tomasz Wasilewski, Zofia Hordyjewicz-Baran

**Affiliations:** 1 Department of Technology of Cosmetic and Pharmaceutical Products, Medical College, University of Information Technology and Management in Rzeszow, Sucharskiego 2, 35-225 Rzeszów, Poland; mzagorska@wsiz.rzeszow.pl (M.Z.-D.); aziemlewska@wsiz.rzeszow.pl (A.Z.); zniziol@wsiz.rzeszow.pl (Z.N.-Ł.); 2 Department of Industrial Chemistry, University of Technology and Humanities in Radom, Chrobrego 27, 26-600 Radom, Poland; tomasz.wasilewski@uthrad.pl; 3 Research and Development Department, ONLYBIO.life Sp. z o.o., Wojska Polskiego 65, 85-825 Bydgoszcz, Poland; 4 ŁUKASIEWICZ Research Network—Institute of Heavy Organic Synthesis “Blachownia”, Energetykow 9, 47-225 Kedzierzyn-Kozle, Poland; zofia.hordyjewicz@icso.lukasiewicz.gov.pl

**Keywords:** plant extracts, natural dyes, cosmetics, biologically active dyes

## Abstract

Nowadays, natural dyes are expected by the cosmetic and food industries. In contrast to synthetic dyes, colorants derived from natural sources are more environmentally friendly and safer for human health. In this work, plant extracts from *Gomphrena globasa* L., *Clitoria ternatea* L., *Carthamus tinctorius* L., *Punica granatum* L. and *Papaver rhoeas* L. as the natural and functional dyes for the cosmetics industry were assessed. Cytotoxicity on keratinocyte and fibroblast cell lines was determined as well as antioxidant and anti-aging properties by determining their ability to inhibit the activity of collagenase and elastase enzymes. In addition, the composition of the extracts was determined. The obtained extracts were also applied in face cream formulation and color analyses were performed. It has been shown that the obtained extracts were characterized by no cytotoxicity and a high antioxidant potential. The extracts also show strong ability to inhibit the activity of collagenase and moderate ability to inhibit elastase and provide effective and long-lasting hydration after their application on the skin. Application analyses showed that the extracts of *P. rhoeas* L., *C. ternatea* L. and *C. tinctorius* L. can be used as effective cosmetic dyes that allow for attainment of an intense and stable color during the storage of the product. The extracts of *P. granatum* L. and *G. globasa* L., despite their beneficial effects as active ingredients, did not work effectively as cosmetic dyes, because cosmetic emulsions with these extracts did not differ significantly in color from emulsions without the extract.

## 1. Introduction

The sustainable products idea is currently present in every aspect of human life. Food, pharmaceuticals, clothing, and other everyday products are produced at an increasing rate in accordance with the principles of sustainable development. The strong need to produce sustainable products is also observed in the cosmetic industry. Due to the growing awareness of consumers and natural cosmetics’ popularity, manufacturers are forced to update their product portfolios. Nowadays, if they want to stay competitive on the market, they must introduce into their offers not only natural cosmetics with high concentrations of naturally derived raw materials, but also sustainable products which, as consumers expect, are combination of a high level of safety for human health and for the environment and high quality, functionality, effectiveness of use as well as maintaining the aesthetic characteristics of the product [1,2,3,4,5,6,7,8,9,10,11].

The production of cosmetics containing raw materials obtained directly from nature in the formulation or high concentrations of naturally derived ingredients does not guarantee a sustainable product. Contrary to the typical natural cosmetics, the development of sustainable cosmetic products requires the use not only of naturally derived raw materials but, above all, effective ingredients that do not have a negative impact on the environment and human health [9,10,11]. An example may be essential oils, which despite, being 100% derived from nature, are characterized by an unfavorable impact on the skin through their high allergic potential. Some essential oils may also be harmful to the environment, especially to the aquatic environment [3,4,5,6]. A similar problem concerns the surfactants used in the formulation of cleansing cosmetics. Despite their natural origin, many of them are characterized by high irritant potential to the skin and have a negative effect on the environment [1,3,7]. In this case, creating a sustainable cleansing cosmetic requires the use of additional ingredients to reduce the negative interactions of the surfactants with the skin or gentle surface active agents with the low irritant potential [12,13,14,15,16,17,18,19]. Unfortunately, both methods produce a significantly higher price of the finished product.

Dyes, which make it possible to obtain the appropriate aesthetic characteristics of the product, are one of the biggest problems for the cosmetics industry in creating sustainable products [2,3,7,8]. In addition to the fragrance, the color of the products is still one of the main criteria used by consumers during cosmetic buying. Most of the dyes commonly used in cosmetics formulations are synthetic, and they are obtained in chemical reactions. In the case of natural cosmetics, manufacturers usually use food dyes with a safer toxicological profile or mineral pigment, such as iron oxides, activated charcoal, chromium oxides or ultramarine [7,8,20]. Some of them, however, are characterized by a negative impact on health through an irritating effect on the skin and eye mucosa. In addition, some coloring agents may adversely affect the natural environment, both as a final product and through the process of their production (by-products of synthesis, high water consumption in the production process, generation of post-production waste) [20]. Therefore, there is a great need to conduct research on new coloring agents for both cosmetics and the food industry, which will be devoid of adverse effects on health and the environment and will comply with the principles of sustainable development.

In this study, an attempt was made to develop environmentally friendly, safe, and functional dyes extracted from plant materials. Such raw materials could be new, biodegradable, multifunctional ingredients of cosmetics which, apart from their dying effect, may also act as bioactive ingredients with antioxidant and anti-ageing potential. In this research, flower extracts derived from five plants rich in natural dyes were analyzed: *Gomphrena globasa* L. (GGE), *Clitoria ternatea* L. (KTE), *Carthamus tinctorius* L. (CTE), *Punica granatum* L. (PGE), *Papaver rhoeas* L. (PRE). The extracts were assessed for their cytotoxicity to skin cells (keratinocytes and fibroblasts), antioxidant potential and the ability to inhibit enzymes responsible for collagen and elastin degradation. The potential of the extracts to reduce transepidermal epidermal water loss (TEWL) and the effect on skin hydration level after their application on the skin was also analyzed. In addition, application analyses were carried out, which included the development of formulations of cosmetic products (face cream containing the obtained extracts as dying and active ingredients). Color analysis was performed for the obtained extracts and cosmetic products.

## 2. Results and Discussion

### 2.1. Determination of Bioactive Compounds

Phenolic compounds included in plant raw materials have special antioxidant and anti-aging properties. Their action is mainly based on the capture of free radicals by the hydroxyl group and the formation of complexes showing much lower reactivity. In addition, biologically active compounds increase the activity of antioxidant enzymes, support the work of repair enzymes, and prevent damage in DNA structures. The flavonoids present in the plant material inhibit the enzyme hyaluronidase, which causes the degradation of hyaluronic acid, and elastase, which leads to the degradation of collagen and elastin fibers. In addition, they have a sealing effect on the capillaries, reduce their permeability and have antiallergic properties [21,22].

Plant extracts are a rich source of biologically active substances. They can significantly affect the condition of the skin, as well as act as auxiliary substances that affect the durability, bioavailability of cosmetic products and its color. The active compounds contained in the analyzed extracts are also characterized by an intense color, which may contribute to shaping the quality of cosmetic products in which the extracts are applied [23,24,25]. During the research carried out in the analyzed plant extracts obtained from *G. globasa* L., *C. ternatea* L., *C. tinctorius* L., *P. granatum* L., *P. rhoeas* L., the antioxidant potential, and the content of biologically active compounds, including phenolic compounds or flavonoids, were assessed.

The obtained HPLC-ESI-MS/MS results in negative-ion mode revealed the presence of bioactive compounds. The structures of detected compounds were confirmed by MS^2^ fragmentation of selected *m*/*z* signals. The investigated extracts were a rich source of polyphenols, of which phenolic acids and flavonoids were the principal compounds. The detected phenolic acids were gallic, caffeic, and chlorogenic acids. Flavonoids were represented by quercetin, rutin and kaempferol derivatives. Table 1 and Table 2 show the bioactive compounds detected by HPLC-ESI-MS/MS in the extracts. The extracted ion chromatograms obtained in negative-ion mode for extracts are presented in a supplementary file.

The determined phenolic content varied among the analyzed extracts. As a result of the LC-ESI-MS/MS comparative analysis of the antioxidant extracts, it was found that gallic acid was the most abundant representative of phenolic acid with the highest content in PGE (13.61 µg/mL). Quercetin representing the group of flavones was found in the highest amount of 16.05 µg/mL in PRE. PRE was the plant with the highest total amount of quantified compounds from the investigated extracts. The dominant polyphenolic acid in it was caffeic acid (1.21 µg/mL). Peaks of kaempferol derivatives were also detected. KTE was found to be a rich plant in all investigated phenolic acids and flavonoids with a total content of bioactive compounds of 15.1 µg/mL. The dominant bioactive compounds in the CTE extract were quinic and caffeic acids. A significant peak for rutin was also detected in CTE. GGE was recognized as a valuable source of biologically active compounds, however with the lowest content of quantified compounds compared to other investigated plant extracts. The total content of quantified active substances was 17.75, 15.12, 15.10, 11.87 and 0.13 µg/mL for the PRE, PGE, KTE, CTE and GGE extracts, respectively.

### 2.2. Determination of Antioxidant Properties

As previously mentioned, phenolic compounds and flavonoids show strong antioxidant properties, as demonstrated by numerous studies. A correlation between the content of bioactive compounds and the antioxidant potential of the extracts has also been demonstrated [26,27]. Detailed analysis of the composition of the examined extracts resulted in detection of, among others phenolic acids, quinic, gallic, caffeic and chlorogenic acids. Flavonoids were represented by quercetin, rutin and kaempferol derivatives [26]. Therefore, in the next step of the research, an analysis of the antioxidant activity of the examined extracts was carried out, where the research was performed using various methods. First, the DPPH method was used. The tests with this method were carried out at a concentration of 100 μg/mL. As a result, seven measurement points were realized for the analyzed concentration of the tested extract.

**Figure 1:**
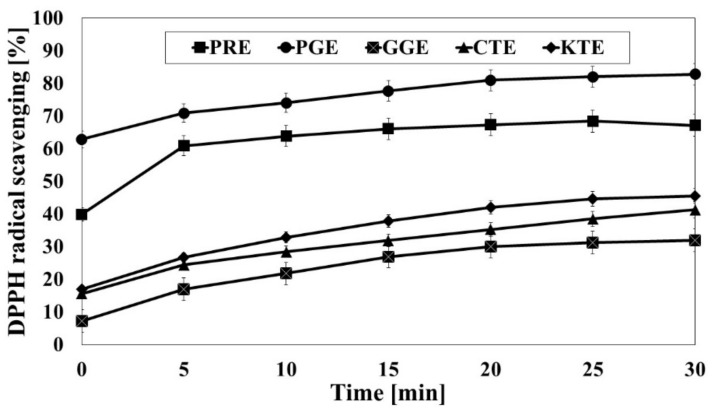
Kinetics of the absorbance changes in DPPH∙ solutions in the presence of 100 μg/mL concentrations of water–ethanol extracts PRE (*P. rhoeas* L.), PGE (*P. granatum* L.), KTE *(C. ternatea* L.), CTE *(C. tinctorius* L.), *GGE (G. globasa* L.). Values are means of three replicate determinations (*n* = 3).

Analysis of Figure 1 leads to the following conclusions. First, in all investigated cases there was a monotonous increase in the DPPH radical scavenging in the time observed. A characteristic finding is that, for each investigated extract, after 30 min of the experiment measured, scavenging was higher by about 20% compared to the initially registered value. Another interesting observation is that, in each moment of the experiment, the investigated extracts are ordered the same way in terms of measured DPPH radical scavenging, starting from the lowest values for the KTE, then successively CTE, GGE, PRE, ending on the highest values for the PGE (Figure 1). The research carried out by Madhu [27] shows an increase in the DPPH with increase in the concentration of the extract, even reaching 70% for the KTE in higher concentration (600 μg/mL).

**Figure 2:**
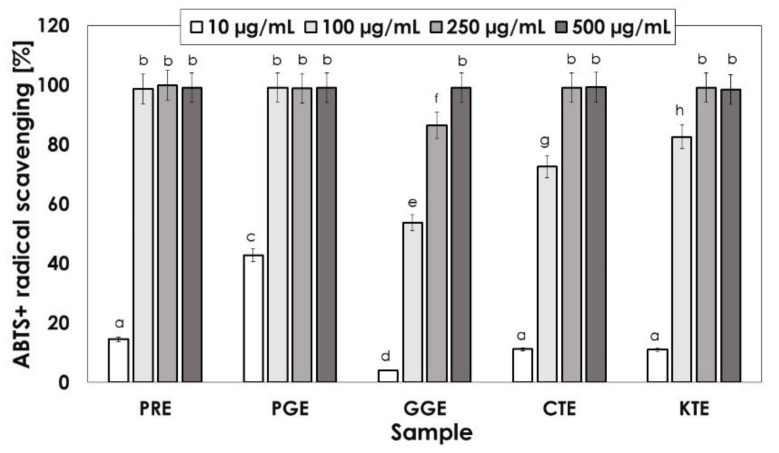
Kinetics of the absorbance changes in ABTS solutions in the presence of various concentrations of water–ethanol extracts PRE (*P. rhoeas* L.), PGE (*P. granatum* L.), GGE (*G. globasa* L.), CTE *(C. tinctorius* L.), KTE *(C. ternatea* L.). Values are means of three replicate determinations (*n* = 3).

Another method was the analysis of the anti-radical activity using the ABTS^. +^ radical. The radical ABTS was scavenged at a concentration of 10–500 μg/mL. First, for all the investigated samples, an increase in scavenging was observed with increasing extract concentration, reaching the same maximum value of 100% at some concentrations of the extract, where the growth is more or less rapid depending on the sample. The maximum scavenging close to 100% is achieved for all the extracts but starting with different concentrations. In the case of the PRE and the PGE, the growth is the fastest. It was observed that, from the concentration of 100 μg/mL, the radical scavenging reaches the maximum value. Then, for the CTE and the KTE, the border concentration is 250 μg/mL, while for the GGE the border concentration is the highest and equals 500 μg/mL (Figure 2).

Free radicals (reactive oxygen species, ROS), generated in the skin cells, are one of the major factors inducing the skin ageing process. Plant extracts as a rich source of antioxidants are able to reduce the intracellular oxidative stress and improve the skin’s power to delay the aging process. Reduction in oxidative stress impacts on acceleration of the skin regeneration which may be important, e.g., in wound healing processes. In our research, the influence of the extracts was analyzed on intracellular production of ROS in the keratinocytes and fibroblasts cells. This study was performed using fluorogenic H_2_DCFDA dye. After passive diffusion of H_2_DCFDA into HaCaT and BJ cells, it is deacetylated by intracellular esterases to a non-fluorescent compound. In the presence of ROS, it is oxidized into highly fluorescent 2′,7′-dichlorofluorescein (DCF).

**Figure 3:**
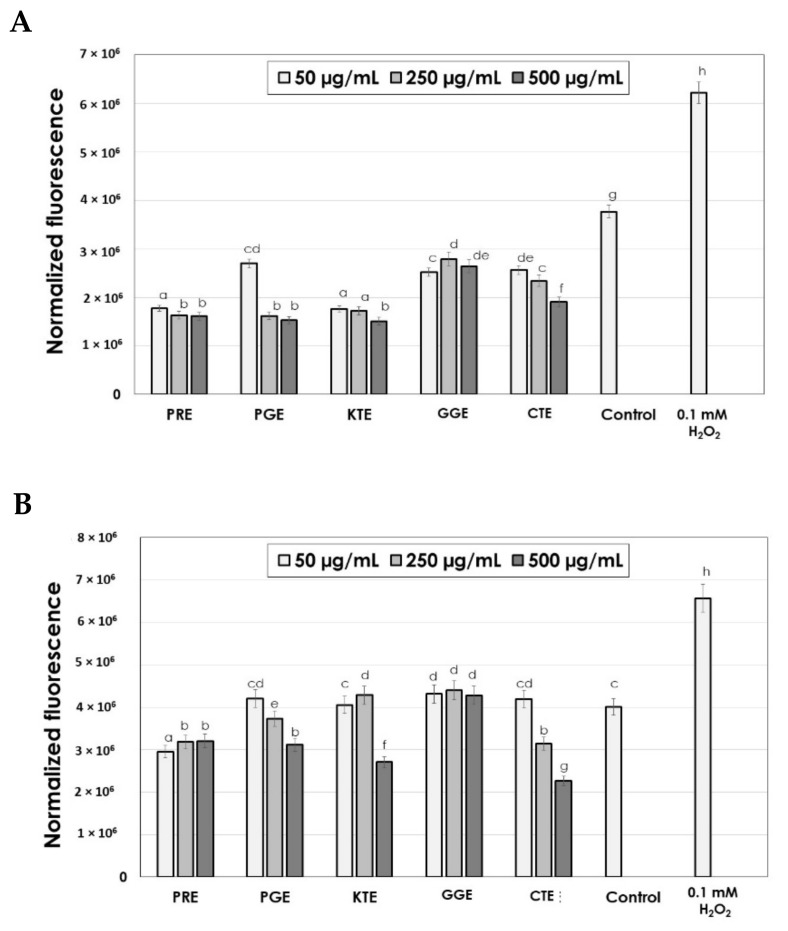
The effect of PRE, PGE, KTE, CTE and GGE on the 2′,7′-dichlorofluorescein (DCF) fluorescence in BJ (**A**) and HaCaT cells (**B**). The data are expressed as the mean  ± SD of 3 independent experiments, each of which consisted of 3 replicates per treatment group.

It was shown (Figure 3A and B) that each of the analyzed extracts has the ability to decrease ROS concentration in BJ cells. The strongest potential to minimize the oxidative stress in fibroblasts was shown for PRE, PGE, CTE and KTE extracts at the concentration of 250–500 μg/mL. Fluorescence for those extracts was about 40–50% lower than for the control (cells not treated with extracts). Intracellular antioxidant properties for GGE extracts were lower than for other extracts and the value of fluorescence was lower about 20–25% in relation to the control. The most effective extracts for lowering ROS level in the keratinocyte cells were the PRE extract (in the concentration range of 50–250 μg/mL) and PGE, KTE and CTE extracts at the highest analyzed concentration of 500 μg/mL. The strong antioxidant potential (two times lower than for the control) was obtained for the CTE extract at the concentration of 500 μg/mL. The GGE extract reached a higher value of fluorescence than the control which may be due to the lowest concentration of active ingredients. Ability to reduce intracellular oxidative stress in HaCaT cells by PGE and KTE extracts at the concentration of 50–250 μg/mL was similar to that of the control.

In this study, the analyzed extracts were obtained from flowers of plants that are rich in natural dyes. The most popular plant dyes belong to the group of flavonoids, of which the largest group is the anthocyanins. *C. tinctorius* L. contains as a major dye carthamin (red–orange dye) [28,29,30], *G. globosa* L. contains betacyanins which are pink–violet in color (gomphrenin, isogomphrenin II, and isogomphrenin III) [31,32,33]. The major dye of *K. terantea* L. flowers are anthocyanins called ternathins [34,35,36]. The color of *P. rhoeas* L. is due to the presence of red in color anthocyanins, whose cyanidol is the main component [37,38]. In addition to dyes, plant extracts contain other active ingredients, including polyphenols, proteins and carbohydrates. Polyphenols and flavonoids are responsible for the antioxidant activity, including the ability to reduce the oxidative stress generated by ROS in cells. Numerous studies have demonstrated strong antioxidant potential of the main coloring compounds contained in plant flowers [28,29,30,31,32,33,34,35,36,37,38,39,40,41]. Slezak et al. have shown that aqueous pomegranate peel extract at low concentrations were characterized by the ability to decrease intracellular (V79 cells) oxidative stress after 24h incubation. With short incubation time (1h), pomegranate extract acts as pro-oxidant [40]. Hong et al. have shown that the addition of Carthamus tinctorius extract as a dye component of food has antioxidant properties and the ability to decrease the intracellular ROS level in HT-29 cells [41]. The analysis of the literature data shows that the other extracts have not been previously tested for their ability to reduce intracellular oxidative stress.

### 2.3. Assessment of Matrix Metallopeptidase Inhibition

**Figure 4:**
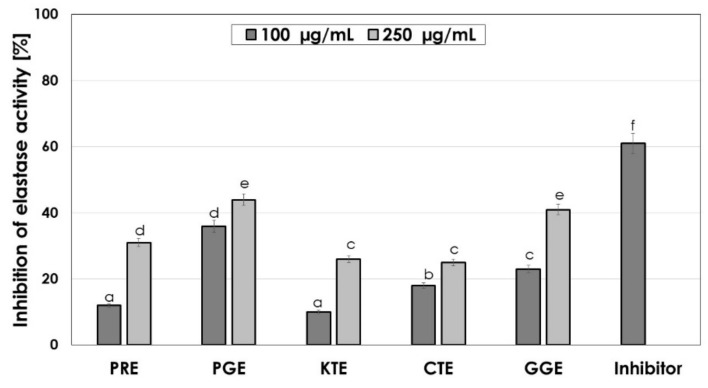
Elastase inhibitory activity of tested plant extracts. Data are the mean of three independent experiments, each consisting of two replicates per treatment group.

**Figure 5:**
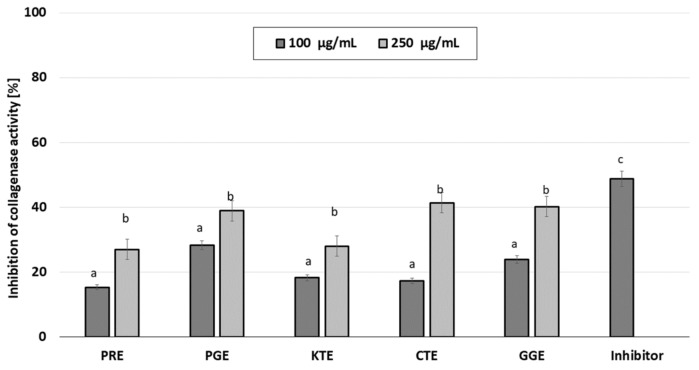
Collagenase inhibitory activity of tested plant extracts. Data are the mean of three independent experiments, each consisting of two replicates per treatment group.

The skin aging process can be divided into two categories: internal and external aging. Internal (natural) aging is caused by changes in skin elasticity over the years. External aging is the result of skin exposure to solar radiation (photoaging). Excessive production of reactive oxygen species resulting from internal or external factors increases the synthesis and activates these proteases, leading to degradation of the extracellular matrix, including collagen and elastin fibers [42]. Eighty percent of the dry weight of the skin is collagen that is responsible for the tensile strength of the skin. Collagen and elastin fibers are produced by fibroblasts and are primarily affected by photoaging, resulting in visible changes in the skin such as wrinkles, pigmentation, and changes in thickness [43]. Plants contain a wide variety of compounds, which may contribute to slowing down the degradation of collagen and elastin contained in the skin and stimulate their synthesis. These properties affect the acceleration of skin regeneration processes, the healing of wounds and scars and the slowing down of skin aging processes [44,45]. The ability to inhibit elastase and collagenase were determined for five types of plant extracts and the results are shown in Figure 4 and Figure 5.

The conducted analysis showed that the tested extracts have different elastase inhibition capacity, which results in inhibition of substrate hydrolysis. In order to compare the relative efficacy of the tested inhibitors, tests with the control inhibitor SPCK were also carried out. It was observed that the inhibitory ability of elastase depends on the concentration of the extract. PGE has the greatest inhibitory properties, being able to inhibit the activity of elastase by almost 50% at a concentration of 250 µg/mL and almost 40% for concentration of 100 µg/mL. Moreover, a high inhibition of the activity of this enzyme (about 40% at a concentration of 250 µg/mL) is observed for GCE. Similar values were obtained for collagenase inhibition. It was observed that the ability to inhibit collagenase also depends on the concentration of the extract. At the concentration of 250 µg/mL, the greatest anti-collagenase activity had PGE, CTE and GGE extract at the level of about 40% and almost 30% for the concentration of 100 µg/mL for PGE. The remaining plants showed a lower inhibition capacity for this matrix metalloproteinase (20–30% for the higher concentration tested). These results suggest that the plant extracts have significant anti-elastase and anti-collagenase properties.

The fact that active ingredients of plant extracts may contribute to the ability for metallopeptidases inhibition has been proven by many authors. Chromatographic analyses of the obtained extracts showed the presence of various polyphenolic compounds such as rutin, quercetin, kaempferol derivatives and phenolic acids such as caffeic, quinic, gallic and chlorogenic (3- and 5-caffeoylquinic acid). Literature studies show that these compounds can prevent collagen and elastin degradation by inhibiting collagenase and elastase activity. A relevant amount of quercetin was noticed in the PRE extract. As far we know, quercetin is a common anti-aging active compound [46]. Quercetin as a lipid peroxidation inhibitor can protect skin from dehydration. Quercetin inhibition of matrix metalloproteinase activity might also show a role in protection of skin collagen from destruction during inflammatory response to extrinsic aging factors [47]. The PGE extract also showed a high content of gallic acid, which may improve skin elasticity in the dermis by scavenging the free radical and inhibiting the MMP-2 activity. Sastravaha et al. have been evidenced that some phenolic compounds, such as tannins present in the PGE extract, can provide a synergistic effect in stabilizing collagen by showing affinity to proteins and thus creating bonds with collagen fibers [48]. The aqueous extracts of KTE flower (250 μg/mL) reduced mtDNA damage in UV-induced human immortalized keratinocytes (HaCaT) [49]. Zemour et al. [50] demonstrated anti-collagenase and anti-elastase activity of CTE oil as a potential raw material for use in cosmetic products. In addition, Dakhil et al. [51] have reported that the properties of CTE oil can make it the main component of the preparations for the treatment of different skin problems. The presence of various polyphenolic compounds may act as promising candidates for anti-aging interventions through suppression nutrient signaling, directly neutralizing stressors, or activating stress-responsive pathways, thereby reducing damage to biomolecules [52]. Moreover, polyphenols can extend lifespan and promote health in various animal models through reduction of oxidative stress and low-grade chronic inflammation, induction of autophagy, as well as regulation of several important molecules involved in enhancing energy homeostasis and mitochondrial function [53]. Due to the significant anti-collagenase and anti-elastase activity of the polyphenols present in the obtained plant extracts, they can be potential candidates for counteracting the effects of aging in the cosmetics and pharmaceutical industries.

### 2.4. Cytotoxicity Assessment

**Figure 6:**
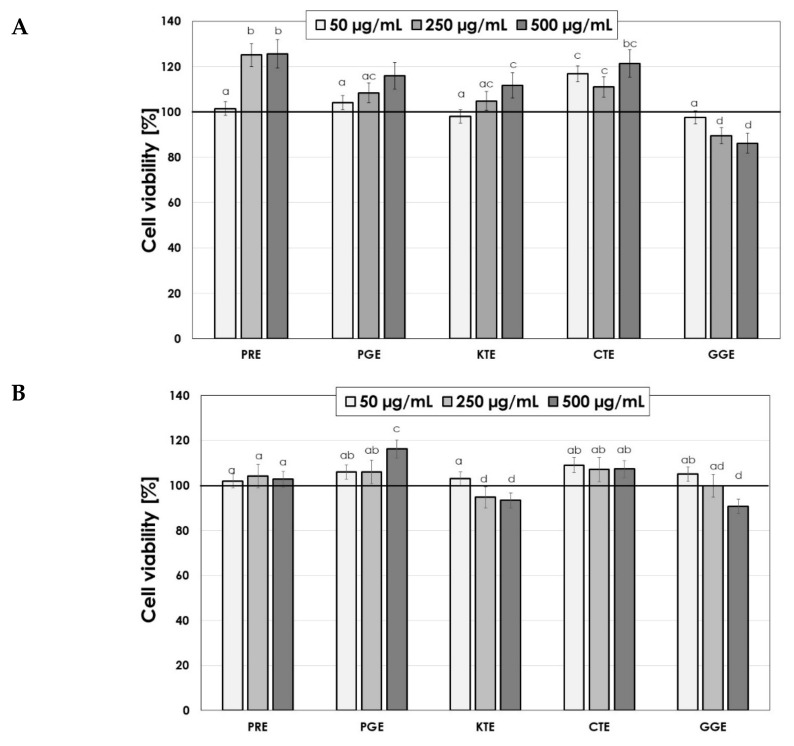
The reduction in resazurin after 24-h exposure to PRE, PGE, KTE, CTE and GGE extracts in cultured fibroblasts (**A**) and keratinocytes (**B**). Data are the mean ± SD of three independent experiments each consisting of four replicates per test group.

**Figure 7:**
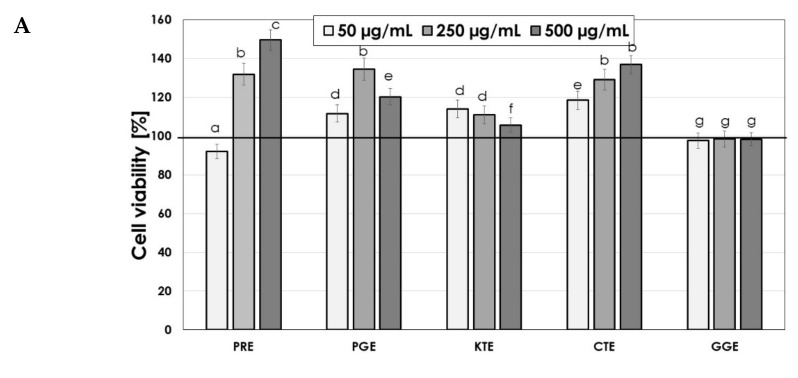

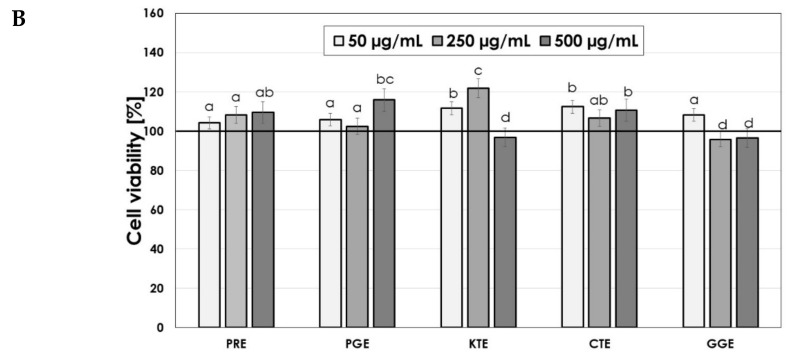
The effect of increasing concentrations of PRE, PGE, KTE, CTE and GGE extracts on Neutral Red Dye uptake in cultured fibroblasts (**A**) and keratinocytes (**B**) after 24 h of exposure. Data are the mean ± SD of three independent experiments each consisting of four replicates per test group.

The assessment of the cytotoxicity of extracts on skin cells is an extremely important element in the context of assessing extracts as potential ingredients of cosmetic preparations. Thus, the next stage of the research was to examine the cytotoxic properties of the tested PRE, PGE, KTE, CTE and GGE extracts. For this purpose, the Alamar Blue and Neutral Red tests were used. The research was carried out on two skin cell lines—fibroblasts and keratinocytes. Resazurin used in the Alamar Blue test is used as an oxidation–reduction indicator, which undergoes a colorimetric change due to the reduction in cellular metabolism, which allows the assessment of cell viability in vitro. Only in the case of the GGE extract was a decrease in the viability of these cells observed after applying higher concentrations of the extract (Figure 6A). The remaining extracts positively influenced the viability of these cells. Similar results were obtained in the case of keratinocytes; however, the cytotoxic effect was also observed in the case of higher concentrations of the KTE extract. The remaining extracts increased the viability of HaCaT cells (Figure 6B). Measurement of the amount of released Neutral Red dye allowed determination of the total number of viable cells treated with the analyzed extracts. In the case of fibroblasts, only the GGE extract did not significantly affect the viability of cells, while the remaining extracts increased the viability by up to 51% in the case of the PRE extract at a concentration of 250 µg/mL. An almost 40% increase was also observed in the case of the use of PGE and CTE extract at concentrations of 250 µg/mL and 500 µg/mL, respectively (Figure 7A). In the case of keratinocytes, all analyzed extracts showed a positive effect on the viability of these cells; however, KTE and GGE extracts at higher concentrations slightly decreased the amount of incorporated dye in lysosomes, which indicates a partial decrease in the activity of these cells. The conducted analysis showed that the tested extracts show a dose-dependent effect on the metabolic activity and proliferation of skin cells (Figure 7B). The lack of cytotoxicity of these extracts indicates that they can be considered as valuable ingredients in cosmetic and dermatological preparations intended for the care and treatment of skin diseases. PRE, PGE and CTE extracts turned out to be particularly promising raw materials.

Due to plant extracts being a valuable source of compounds with valuable biological properties, they are often perceived as valuable raw materials with a positive effect on cells, including skin cells. However, it is worth noting that some of the compounds present in plants exhibit cytotoxic effects, and hence the analysis of the effect of these extracts on cell viability is extremely important to assess the possibility of their use in various types of preparations [54,55]. In particular, there are only a few scientific reports regarding the cytotoxicity of the plants we tested, especially in the context of the skin. The cytotoxicity of the compounds contained in PRE has previously been studied by other authors, but most of the research has focused on the analysis of the effect on cancer cells. The effect of alkaloids contained in PRE was studied by Ali Hijazi et al. using a variety of cell lines, both normal and cancerous. These authors showed that the IC50 values for individual alkaloids on normal cells were higher than on cancer cell lines, which indicates the selectivity of the cytotoxic activity of these compounds towards cancer cells. These authors also indicated that, despite the cytotoxic effect of selected alkaloids from PRE on keratinocytes, the extract itself shows much lower cytotoxic effects [56]. This is probably due to the presence of other biologically active compounds in PRE extracts, such as, inter alia, phenols and flavonoids, which positively affect the viability of keratinocytes and thus result in the fact that the entire extract increases metabolic activity and cell proliferation, as shown in our research analyses. The positive effect of PGE extracts has already been shown by other authors who indicated that both the type of extract and the part of the plant affect the properties of the obtained extract. Aslam and colleagues pointed out that pomegranate peel extract primarily supports the regeneration of the dermis, while pomegranate seed oil mainly supports the regeneration of the epidermis [57]. Pacheco-Palencia et al. demonstrated the protective effect of PGE against damage to skin fibroblasts caused by UVA and UVB cells. These authors indicated that the effect of this extract was probably related to the decreased activation of the pro-inflammatory transcription factor NF-kappaB, the pro-apoptotic caspase-3 and the increased G0/G1 phase, which resulted in the repair of DNA damage [58]. Nasiri et al. however, indicated that PGE flower extracts can stimulate the proliferation of skin cells and support wound healing processes in vivo [59]. The positive effect of KTE on the proliferation of keratinocytes and fibroblasts was also previously indicated by Zagórska-Dziok et al., who did not observe any cytotoxic effect and damage to the cell membranes treated with extracts from flowers of this plant [60]. Additionally, Zakaira et al. showed a positive effect of KTE flowers on keratinocytes and indicated a protective effect against hydrogen peroxide-induced cytotoxicity. This effect was probably the result of the antioxidant activity of polyacylated anthocyanins and flavonol glycosides contained in the KTE flower extract [49]. CTE extracts have also been the subject of scientific research that indicates their valuable properties. Junlatat et al., in their research, indicated that CTE floret extract stimulates the proliferation of both dermal papilla cells and keratinocytes (HaCaT) and can significantly stimulate vascular endothelial growth factor and keratinocyte growth factor [61]. Moreover, the studies of Liu et al. showed that higher concentrations of CTE can inhibit the proliferation and synthesis of collagen by fibroblasts in a hypertrophic scar in vivo, which may contribute to the inhibition of this type of scar formation [62]. GGE flower extract has not been tested for cytotoxicity to skin cells so far, so this work for the first time indicates the possibility of safe use of this plant extract in preparations intended for contact with the skin. The positive effect of the analyzed extracts is certainly related to the presence of numerous biologically active compounds, the interaction of which contributes to the protective effect of these plants on the cells of various layers of the skin.

### 2.5. Application Analysis

#### 2.5.1. Influence of the Extracts on the Skin Condition and Sun Protection Factor (SPF)

**Figure 8:**
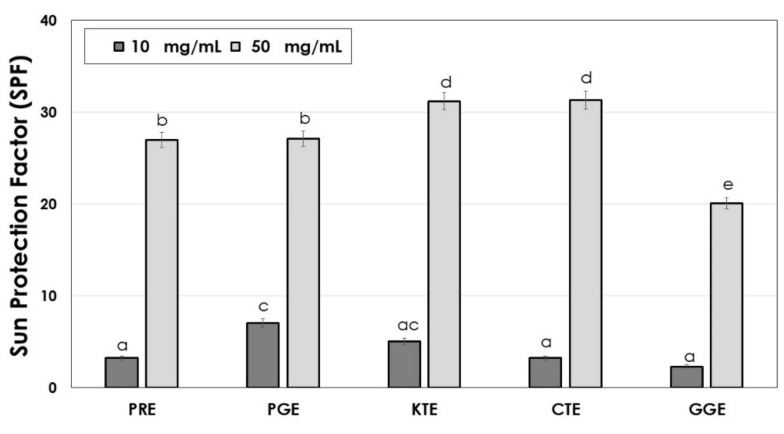
Ability of PRE, PGE, KTE, CTE and GGE extracts to protect against UV radiation.

Plant extracts are the source of many active ingredients able to absorb UV radiation. Active ingredients of plant extracts that give them natural sunscreen properties are mainly flavonoids and polyphenols, as well as proteins, amino acids, and vitamins. Anthocyanins are the group of bioactive substances that are indicated as strongest natural UV filters [63,64,65]. Extracts rich in plant dyes can therefore be effective natural sunscreens [63,64,65,66,67,68]. Sun protection factors (SPF) analysis was carried out for the obtained extracts at the concentrations of 10 and 50 mg/mL. The results are shown in Figure 8.

It was shown the analyzed plant extracts are characterized by high SPF factors. At the concentration of 50 mg/mL, the KTE and CTE extracts reach the highest SPF value (SPF of about 31) due to the strong ability to absorb UV radiation. Slightly lower values (SPF of about 28) were demonstrated for the PRE and PGE extracts and the lowest SPF value (SPF of about 20) was observed for the GGE extract. Korać and Khambholia indicated that active ingredients of plants with the greatest impact on the SPF are quercetin, cyanidin, apigenin, caffeic, quinic and ferulic acid, as well as silymarin, resveratrol, juglone, ascorbic acid, tocopherols, and carotenoids [65]. Ranjithkumar et al. showed that Pomegranate seed oil has a strong ability to absorb the UV radiation at the level of synthetic sunscreens (SPF ca. 20) and pomegranate fruit and peel extracts prepared in methanol may act as UV boosters, increasing the SPF value of the synthetic and mineral UV filters [66]. Westfall et al. showed that lipsticks pigmented by naturally derived anthocyanins achieve a SPF higher than 15 [64].

**Figure 9:**
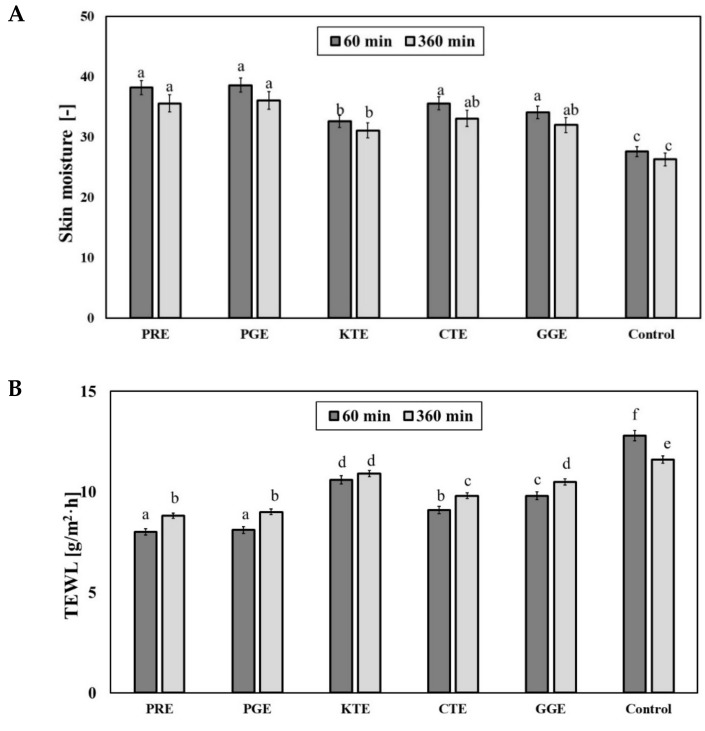
Influence of PRE, PGE, KTE, CTE and GGE extracts on skin hydration (**A**) and TEWL (**B**). Different letters on the charts indicate significant differences between groups (*p <* 0.05).

Bioactive ingredients of plant extracts also positively affect the condition of skin by increasing its moisture. Substances like flavonoids and polyphenols as well as proteins and amino acids, due to the presence of hydroxyl groups in the molecules, may form hydrogen bonds with water and hold moisture in the skin. It impacts not only the skin moisture level but also may lower the amount of water that evaporates from the upper layer of epidermis (transepidermal water loss, TEWL) [69]. For the obtained extracts, the effect on the skin moisture and TEWL was determined after their application on the skin (Figure 9A,B).

The application of the analyzed extracts (concentration of 10 mg/mL) on the skin causes a significant increase in skin moisture level. A total of 60 min after extract application, PRE, PGE, CTE and GGE extracts showed the strongest moisturizing properties. In relation to the control field (without extract application), a rise in the analyzed parameter by about 20–30% was noted. KTE extract showed slightly weaker moisturizing properties. A total of 360 min after application of the analyzed extracts on the skin not significant decrease in skin moisture was noted, but the observed values were significantly different from the control. The skin moisture level observed for PRE, PGE, CTE and GGE extracts was about 25–35% higher than for the control. In the case of KTE extract, an increase in the skin moisture by about 10% was observed (versus the control). Similar results were obtained in the TEWL analysis. The strongest properties related to TEWL decrease were observed for PGE and PRE extracts. TEWL value 60 min and 360 min after application of those extracts was lower in relation to the control field by about 35% and 25%, respectively. For the KTE extract, decrease in TEWL was about 15% and 5% in relation to the control, respectively, after 60 and 360 min from the moment of the extract application. The obtained results indicate that the analyzed extracts provide long-lasting skin moisture properties and are effective protectants against excessive water loss.

#### 2.5.2. Determination of the Color Parameters of Extracts

The active compounds contained in plant extracts can be used as dyes. The analyzed extracts were subjected to colorimetric tests (Table 3).

Based on the experiment, it was found that, among the examined extracts, the following samples have the highest potential for use as a dye: *P. rhoeas* L. (PRE), *C. ternatea* L. (KTE) and *C. tinctorius* L. (CTE). For PRE and CTE, relatively high chroma values (C*) were obtained, 3.7 and 8.2, respectively. Based on the values of the h^o^ parameter, it was found that the clearly discernible colors of the above extracts are red and yellow, respectively. An interesting extract, from the point of view of the possibility of dyeing cosmetics, is also the one marked as KTE (blue–violet color). Although the C * value for KTE was relatively low (2.2), the color of the extract was clearly visible to the naked eye. The extracts of *P. granatum* L. (PGE) and *G. globasa* L. (GGE) were yellow in color, and the obtained chroma values were at the level of 2.5. For this color, these values were not significantly discernible to the naked eye. The use of the last of the plants mentioned as potential dyes requires the use of much higher concentrations.

#### 2.5.3. Determination of the Color Parameters of Cosmetics Based on the Extracts

**Figure 10:**
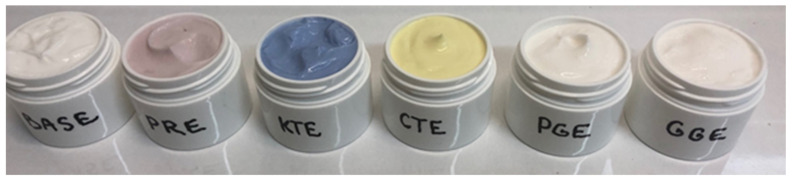
Photo of model face skin care creams containing water–ethanol extracts: *P. rhoeas* L. (PRE), *P. granatum* L. (PGE)*, C. ternatea* L. (KTE), *C. tinctorius* L. (CTE)*, G. globasa* L. (GGE).

Plant extracts rich in bioactive substances, which additionally have the ability to impart color, can be a valuable raw material for the cosmetics industry [70]. In this study, an attempt was made to demonstrate the empirical possibility of using the developed extracts in cosmetics. A series of facial skin care creams containing 1% of the analyzed extracts was prepared (Figure 10). Detailed results related to the marked and calculated color parameters for model creams are presented in Table 4.

It was found that the addition of 1% water–ethanol extract of *P. rhoeas* L. (PRE), *C. ternatea* L. (KTE) and *C. tinctorius* L. (CTE) significantly modifies the appearance of the cream. The individual preparations had a clearly visible color, respectively: red, blue–violet and yellow. Confirmation of the possibility of using the above-mentioned extracts as potential dyes are relatively high values of ΔE_cream+extract/base cream_, related to the color change of the cream with the addition of the extract compared to the base cream. Literature data [71] indicate that, in the case of the value of DE > 5, the recipient perceives individual colors as completely different. For creams with PRE, KTE and CTE extracts, the values obtained were, respectively: 11.88; 31.71 and 22.72. In the case of creams with extracts: *P. granatum* L. (PGE) and *G. globasa* L. (GGE), no significant effect of the extract on the color of the preparation was observed. The values of DE are in the range 1.59–1.63. This means that the difference in color is only noticeable for an experienced observer [70,71].

## 3. Materials and Methods

### 3.1. Plant Material and Extraction Procedure

Plant material (*G. globasa* L., *C. ternatea* L., *C.tinctorius* L., *P. granatum* L., *P. rhoeas* L. dry flowers) was purchased from a local herbal store. All plant flowers were collected on controlled and ecological plantations. The extraction process was carried out with ultrasound according to the method described by Yang et al. [21] in an ultrasonic bath (Digital Μgtrasonic Cleaner, Berlin Germany) equipped with a time controller. The extracts were prepared by extracting 10 g of dry flowers in 100 g water–ethanol solution (80:20). The mixtures were extracted at room temperature for 20 min. When the extract temperature reached 25 ℃ the extract was rapidly cooled with ice to 22–23 ℃. The obtained extracts were then collected and filtered three times through Whatman No. 1 filter paper using vacuum filtration. After filtration, the extracts were evaporated under reduced pressure at 40 °C A stock solution at the concentration of 100 mg/mL was prepared from the dried extracts and was stored in the dark at 4 °C until further analysis. The following abbreviations are used: PRE—Papaver rhoeas extract, PGE—Punica granatum extract, KTE—Clitoria ternatea extract, CTE—Carthamus tinctorius extract, GGE—Gomphrena globasa extract.

### 3.2. Determination of Bioactive Compounds and Antioxidant Properties

#### 3.2.1. Determination of Bioactive Compounds by HPLC–UV-ESI–MS

The obtained extracts were analyzed to determine their main bioactive compounds using high-performance liquid chromatography, HPLC (DionexΜgtiMate 3000 RS Thermo Fisher Scientific, Sunnyvale, CA, USA), coupled to mass spectrometer, MS (4000 QTRAP, AB Sciex, Concord, ON, Canada), equipped with an electrospray ionization source (ESI) and a triple quadrupole-ion trap mass analyzer, working in the multiple reaction monitoring (MRM) scan mode. Chromatographic separation was achieved with a gradient reverse-phase system. A 100 × 4.6 mm chromatographic column Kinetex 3.5 µm XB-C18 100 Å with iso-butyl side chains and with a TMS endcapping stationary phase, used with s similar composition guard column, was purchased from Phenomenex and maintained at 30 °C. A binary solvent system comprising 0.1% (*v*/*v*) aqueous formic acid as solvent A and methanol as solvent B was used under gradient mode for 19.1 min of the run time. The elution conditions applied were as follows: 0.0–15.0 min 25–100% B, 15.0–17.0 min 100% B, 17.0–17.1 min 100–25% B, 17.1–19.1 min 25% B. The flow rate of the mobile phase was 0.6 mL/min and injection volume 10 μL. The eluent was monitored by an electrospray ion mass spectrometer (ESI-MS) under negative ion mode and scanned from *m*/*z* 20 to 1000 Da. For quantification analysis, the triple quadrupole MS detector was working in multiple reaction monitoring (MRM) scan mode. Optimal mass analyzer conditions and the selection of product ions for individual compounds were determined experimentally. For this purpose, standard solutions of investigated compounds (1 ng/mL) in mobile phase composition were introduced using an infusion pump operating in constant sample delivery. After ensuring that the correct precursor ion was selected declastering potential (DP), entrance potential (EP), collision cell exit potential (CXP), collision energy (CE) were optimized for each MRM transition (Figure S1). Two MRM transitions were monitored, one for quantification and one for confirmation. The MS parameters were set as follows: capillary temperature of 600 °C, curtain gas at 35 psi, nebulizer gas at 60 psi and drying gas at 50 psi. A negative ionization mode source voltage −4500 V was applied for determination of bioactive compounds. Nitrogen was used as the curtain and collision gas. Data analysis was processed with Analyst 1.5.1 software. The identification of selected compounds was conducted by molecular mass and fragment of anion entries of each individual compound and confirmed by MS2 fragmentation. The identities of 9 compounds were determined along with their chemical formula, deprotonated molecular ions and the characteristic fragment ions for each individual peaks. A total of 6 compounds were quantified based on the calibration curve generated using peak areas of the most intense MRM transitions of analytical standards. The linearity of the detector response for quantified compounds was demonstrated by injection of calibration standards at eight concentration levels ranging from 0.1 μg/mL to 20 μg/mL. Calibration curves were linear with the coefficients of correlation (R) being greater than 0.99. In cases where the samples did not fall in the linear range of the MS detector, the samples were diluted. Analytical standards of quinic acid, gallic acid, caffeic acid, caffeoylquinic acids (CQA, two isomers: 3- and 5-CQA) and quercetin were purchased from Sigma-Aldrich, Saint Louis, MO, USA). All standards used were of analytical grade (≥99% purity). Standard stock solutions were prepared by accurately weighing and dissolving 20 mg of each standard in 10 mL LC-MS grade methanol to give a concentration of 2 mg/mL. Serial dilutions of 2.0 μg/mL, 1.5 μg/mL, 1.0 μg/mL, 0.5 μg/mL, 0.1 μg/mL, 0.05 μg/mL, 0.02 μg/mL, and 0.01 μg/mL were then made using LC-MS grade methanol solution. The LC-MS/MS assay was performed in triplicate. Obtained data were presented as means ± standard deviations. 

#### 3.2.2. DPPH Radical Scavenging Assay

The ability of the obtained extracts to scavenge free radicals was determined using the 1,1-diphenyl-2-picrylhydrazyl (DPPH) radical. The method described by Brand-Williams et al. [72] was used. Initially, 33 µL of aqueous solutions of extracts at concentrations of 100 µg/mL were mixed with 167 µL methanol solution of DPPH (4 mM) and transferred to a 96-well plate. The analyzed samples were thoroughly mixed by shaking. In the next step, the absorbance of the samples at 517 nm was measured. Measurements were made every 5 min for 30 min on a UV-VIS Filter Max λ = 5 spectrophotometer (Thermo Fisher Scientific, Waltham, MA, USA). Three independent replicates were performed for each extract. Water with a DPPH solution was used as a control. The antioxidant capacity was expressed as a percentage of DPPH inhibition using the equation:

(1) $$\%\;\mathrm{DPPH}\;\mathrm{scavenging}=\frac{Abs\;control-Abs\;sample\;}{Abs\;control\;}\;\times \;100$$

where: Abs control is the absorbance of the control sample (containing DPPH and water), Abs sample is the absorbance of the test sample (containing DPPH and test sample).

#### 3.2.3. ABTS+ Scavenging Assay

Scavenging of the ABTS + (2,20 -Azino-bis(3-ethylbenzothiazoline-6-sμgfonic acid) diammonium salt) free radical was evaluated according to procedure described by Gaweł-Beben et al. [73]. Then, 19.5 mg ABTS and 3.3 mg potassium persulfate were mixed with 7 mL phosphate buffer (pH = 7.4) and dissolved for 16 h in darkness. The solution was diluted to the absorbance at the level of about 1.0 (measurement at λ = 734 nm). Then, 20 µL of KTE, PGE, PRE, CTE and GGE extracts was mixed with 980 µL of diluted ABTS•+ solution and then incubated for 10 min in darkness. The decrease in ABTS•+ absorbance was measured at λ = 734 nm using a UV/VIS spectrophotometer Aquamate Helion (Thermo Fisher Scientific, Waltham, MA, USA). Distilled water was used as a blank. The ABTS + scavenging was calculated from the equation:

(2) $$\%\;\mathrm{of}\;{\mathrm{ABTS}}^{•}+\mathrm{scavenging}=1-\frac{As}{Ac}\;\times \;100$$

where: As—absorbance of the sample; Ac—absorbance of the control sample. Measurements were carried out in triplicate for each extract sample.

#### 3.2.4. Detection of Intracellular Levels of Reactive Oxygen Species (ROS)

To determine the ability of the analyzed extracts (PRE, CTE, GGE, KTE and PGE) to generate the intracellular production of reactive oxygen species in HaCaT and BJ cells, a fluorogenic H2DCFDA dye was used. After passive diffusion of this compound into the cells, it is deacetylated by intracellular esterases to a non-fluorescent compound. In the presence of reactive oxygen species, it is oxidized and transformed into highly fluorescent DCF. To determine the intracellular level of ROS in HaCaTs and BJ, cells were seeded in 96-well plates at a density of 1 × 104 cells per well. Then, cells were cultured in an incubator for 24 h. DMEM medium was removed and replaced with 10 µM H2DCFDA (Sigma Aldrich, Saint Louis, MO, USA) dissolved in serum free DMEM medium. HaCaT and BJ cells were incubated in H2DCFDA for 45 min and then incubated with the extracts in the concentration range of 50–500 µg/mL. Cells treated with 1 mM hydrogen peroxide (H2O2) were used as positive controls. The control samples were cells untreated with the tested extracts. DCF fluorescence was measured every after 90 min using a FilterMax F5 microplate reader (Thermo Fisher Scientific) at a maximum excitation of 485 nm and emission spectra of 530 nm [74].

### 3.3. Assessment of Matrix Metallopeptidases Inhibition

#### 3.3.1. Determination of Anti-Elastase Activity

To determine the possibility of inhibiting matrix metalloproteinase, neutrophil elastase (NE), a fluorometric kit (Abcam, ab118971) was applied. According to the manufacturer’s instructions and with the procedure described previously by Nizioł-Łukaszewska et al. [75], analyses were performed in a standard 96-well plate with a clear flat bottom. Analysis was carried out for all plant extracts in concentrations of 100 and 250 µg/mL. Initially, NE enzyme solutions, an NE substrate and an inhibitor control (SPCK) were prepared according to the instructions. Then, diluted NE solution was added to all wells. Test samples, the inhibitor control and the enzyme control (Assay Buffer) were added to subsequent wells. All samples were prepared in duplicate. After all reagents were added, the samples were mixed. The plate was then incubated at 37 °C for 5 min. In the meantime, a reaction mixture was prepared by mixing the Assay Buffer and NE substrate. The mixture was added to each well and mixed thoroughly. Fluorescence was measured immediately at excitation wavelength λ = 400 nm and emission λ = 505 nm using a microplate reader (FilterMax F5, Thermo Fisher Scientific, Waltham, MA, USA). The kinetic mode was used (30 min at 37 °C). The ability to inhibit NE activity of the analyzed samples was calculated from the equation:

(3) $$\%\;\mathrm{relative}\;\mathrm{NE}\;\mathrm{activity}=\frac{∆\mathrm{RFU}\;\mathrm{test}\;\mathrm{inhibitor}\;}{∆\;\mathrm{RFU}\;\mathrm{Enzyme}\;\mathrm{control}}\;\times \;100$$

The final result was the arithmetic mean of three independent measurements.

#### 3.3.2. Determination of Anti-Collagenase Activity

To assess the ability of the obtained extracts from PRE, PGE, KTE, CTE and GGE to inhibit collagenase activity, a fluorometric kit (Abcam, ab211108) was applied. According to the manufacturer’s instructions and with the procedure described previously by Nizioł-Łukaszewska et al. [75], analyses were performed in a standard 96-well plate with a clear flat bottom. The analysis used analogous concentrations of the tested samples as in the case of the test described above, evaluating the possibility of elastase inhibition. Initially, collagenase (COL) was dissolved in a collagenase analysis buffer (CAB). Test samples were prepared by adding the analyzed samples to COL and CAB. Inhibitor control samples were prepared by mixing the collagenase inhibitor (1,10-phenanthroline (80 mM)) with collagenase and CAB buffer. Enzyme control wells were prepared by mixing diluted COL with CAB. The CAB buffer was used as a background control. The prepared samples were incubated for 15 min at room temperature. In addition, a reaction mixture was prepared by mixing the collagenase substrate with CAB. The reaction mixture prepared in this way was added to all analyzed samples and mixed thoroughly. In the next step, fluorescence was measured at excitation wavelength λ = 490 nm and emission λ = 520 nm using a microplate reader (FilterMax F5, Thermo Fisher Scientific, Waltham, MA, USA). The measurement was performed in kinetic mode for 60 min at 37 °C. All samples were prepared in duplicate according to the manufacturer’s instructions. The ability to inhibit COL activity of obtained extracts was calculated by the equation:

(4) $$\%\;\mathrm{relative}\;\mathrm{COL}\;\mathrm{inhibition}=\frac{\mathrm{enzyme}\;\mathrm{control}-\mathrm{sample}}{\mathrm{enzyme}\;\mathrm{control}}\;\times \;100$$

### 3.4. Cytotoxicity Analysis

#### 3.4.1. Cell Culture

Two skin cell lines were used in the experiments conducted as part of this work. The first were HaCaT cells (normal human keratinocytes) purchased from the CLS Cell Lines Service (Eppelheim, Germany), while the second were BJ cells (fibroblasts, ATCC^®^CRL-2522 ™) obtained from the American Type Culture Collection (Manassas, VA, USA). Cultured cell lines were maintained in DMEM (Dulbecco’s Modification of Eagle’s Medium, Biological Industries, Cromwell, CO, USA) supplemented with L-glutamine, 4.5 g/L glucose and sodium pyruvate. According to the recommendations, the medium was additionally enriched with 10% (*v*/*v*) fetal bovine serum (FBS, Gibco, Waltham, MA, USA) and 1% (*v*/*v*) with antibiotics (100 U/mL penicillin and 1000 µg/mL streptomycin, Gibco) which were added to prevent microbial contamination of the cell culture. Cells were grown in an incubator at 37 °C in a humidified atmosphere of 95% air and 5% carbon dioxide (CO_2_).

#### 3.4.2. Assessment of Cytotoxicity of Tested Extracts

After the cultured cells (HaCaT and BJ) had reached the desired confluence, the DMEM medium was aspirated in the culture flasks (VWR, Radnor, PE, USA) and the bottom-attached cells were washed twice with sterile PBS (phosphate buffered saline, Gibco). The cell layer was detached with trypsin/EDTA (Gibco) and then the cells were placed in fresh DMEM medium. The cells were then seeded in 96-well flat bottom plates (VWR, Radnor, PE, USA). After attaching HaCaT cells and fibroblasts to the bottom of the plates, the cells were treated with PRE, PGE, KTE, CTE and GGE extracts at concentrations of 50, 250 and 500 μg/mL. Cells were then cultured in an incubator for 24 h. The controls were cells (separately HaCaT and fibroblasts) grown in DMEM medium without the addition of test extracts.

#### 3.4.3. Alamar Blue Assay

The first test used to evaluate the cytotoxicity of the tested PRE, PGE, KTE, CTE and GGE extracts and their effect on metabolic activity and viability was the Alamar Blue assay (Sigma, R7017, Life Technologies, Bleiswijk, The Netherlands). The tests were performed according to the procedure described by Zagorska-Dziok et al. [60]. Briefly, after a 24-h exposure of HaCaT and BJ cells to the analyzed extracts in the concentration range of 50–500 µg/mL, a resazurin solution with a concentration of 60 µM was added to each well. The plates were then kept in an incubator at 37 °C for 2 h. After incubation, fluorescence measurements were performed at λ = 570 nm using a FilterMax F5 microplate reader (Thermo Fisher Scientific, Waltham, MA, USA). Three independent experiments were carried out in the study, in which each concentration was tested in four replications. The results are presented as the percentage of cell viability compared to the control cells that were not treated with the extracts (100%).

#### 3.4.4. Neutral Red Uptake Assay

The second test used to evaluate the cytotoxicity of the analyzed extracts was the Neutral Red Uptake Assay (Sigma Aldrich). The research was carried out according to the procedure previously described by Repetto et al. [76]. After plating both cell types in 96-well plates (10^4^ cells per well) and 24-h incubation, the DMEM medium was removed and replaced with PRE, PGE, KTE, CTE and GGE extracts dissolved in DMEM medium. The analyses used extracts at concentrations of 50, 250 and 500 μg/mL. After 24 h of exposure of the cells to the test extracts, they were aspirated and replaced with neutral red dye (40 µg/mL) dissolved in serum-free medium (DMEM) and incubated for 2 h. The cells were then washed twice with phosphate buffered saline (PBS), then 150 μL of decolorizing buffer (EtOH/AcCOOH/H_2_O, 50% (*v*/*v*)/1% (*v*/*v*)/49% (*v*/*v*) was added to each well to extract neutral red retained inside fibroblast and keratinocyte cells. The uptake of neutral red dye was determined by measuring the optical density (OD) at λ = 540 nm in a FilterMax F5 microtiter plate spectrophotometer (Thermo Fisher). The analyses were performed in three independent experiments in which each sample was examined in four replications. The results are presented as a percentage of the control value which is the optical density of cells untreated with the extracts (100%).

### 3.5. Transepidermal Water Loss (TEWL) and Skin Hydration Measurements

TEWL and skin hydration measurements were conducted using a TEWAmeter TM 300 probe and Corneometer CM 825 probe connected to an MPA adapter (Courage + Khazaka Electronic, Köln, Germany). The study was carried out on 15 volunteers. Six areas (2 × 2 cm in size) were marked on the forearm skin of each volunteer. An amount of 0.2 mL of PGE, PRE, KTE, GGE and CTE extracts at a concentration of 100 µL mL was applied to 5 fields. One field (control field) was not treated with any sample. The samples were gently spread into each field and left for 30 min. After 60 and 360 min, the hydration level and TEWL measurements were taken. The final result was the arithmetic mean (from each volunteer) of 5 independent measurements (skin hydration) and 20 measurements (TEWL).

### 3.6. Determination of Sun Protection Factor (In Vitro)

The sun protection factor (SPF) of green coffee extract and fermented green coffee was determined according to the method described by the Mansur Equation. SPF was determined by measuring the absorbance of aqueous solution (50 µg/mL) of the dried extract or the ferment within the wavelength range from 290 to 320 nm at 5-nm intervals. SPF was calculated from the Mansur Equation [77]:

(5) $$\mathrm{SPF}=\mathrm{CF}\;\times \;\sum_{290}^{320}\left[\;\mathrm{EE}\;\left(\lambda \right)\;\times \;I\left(\lambda \right)\;\times \;\mathrm{ABS}\;\left(\lambda \right)\;\right]$$

where: EE (λ)—erythemal effect spectrum, I (λ)—solar intensity spectrum, Abs (λ)—absorbance of sunscreen product, CF—correction factor (= 10), E (λ) × I(λ)—values determined by Sayre were used [78].

### 3.7. Preparation of Model Cosmetics Containing Extracts

A model cream for facial skin care was prepared. All the components used were in line with EcoCert and COSMOS requirements. The formulation is shown in Table 5.

The components of the hydrophobic phase (items 1 to 6) and the hydrophilic phase (items 7–9) were placed in separate beakers, heated to a temperature of 75 °C and thoroughly mixed. The phases were then combined and stirred until cooled to room temperature. After cooling, the preservative (item 10) was added to the cream and mixed thoroughly. In the last step, the pH of the formulation was adjusted. The cream was divided into portions. An amount of 1% of the stock solutions of the extracts was added to each portion and mixed thoroughly.

### 3.8. Determination of the Color Parameters of Extracts and Cosmetics Containing Extracts

Samples of extracts and creams with extracts were tested at room temperature, 48 h after their preparation. A CHROMA METER CR-400 (Konica Minolta, Sensing Inc., Japan) was used to evaluate the color parameters (CIELAB coordinates). The CIELAB system was defined by the International Commission on Illumination in 1978. It is based on three color attributes: L*, a*, b*, where L* is a brightness variable proportional to the value in the Munsell system, and a* and b* are chromatic coordinates. The a* and b* coordinates indicate positions on the red/green and yellow/blue axes, respectively (+a = red, −a = green; +b = yellow, −b = blue).

Based on the data obtained: L*, a* and b*, the following color parameters were calculated: chroma (C*) and hue (*h^O^*). The following equations were used:

(6) $$C^{*}=\sqrt{{\left(a^{*}\right)}^{2}+{\left(b^{*}\right)}^{2}}$$

(7) $$h^{o}=arctan\frac{b^{*}}{a^{*}}$$

General color difference (E cream+extract/base cream) was calculated according to the following formula:

(8) $${∆E}_{\mathrm{cream}+\mathrm{extract}/\;\mathrm{base}\;\mathrm{cream}}^{*}=\sqrt{{\left({∆L}^{*}\right)}^{2}+{\left({∆a}^{*}\right)}^{2}+{\left({∆b}^{*}\right)}^{2}}$$

where: where: ∆L*, ∆a*, and ∆b* are the mathematical differences between creams with extracts L*, a*, b* and base cream L*, a*, b* values. 

### 3.9. Statistical Analysis

Values of different parameters were expressed as the mean ± standard deviation (SD). The two-way analysis of variance (ANOVA) and Bonferroni posttest between groups were performed at the level *p* value of <0.05 to evaluate the significance of differences between values. Statistical analyses were performed using GraphPad Prism 8.4.3 (GraphPad Software, Inc., San Diego, CA, USA) and Statistica 9.0 (StatSoft, CA, USA) using one-way ANOVA and Tukey’s test.

## 4. Conclusions

The research results presented in this work indicate that the studied extracts can be perceived as a valuable source of safe and functional dyes that can be widely used in the cosmetics industry. The undeniable advantage of these raw materials is the wide spectrum of biological activity that has been proven in this work, thanks to which they can not only be an ingredient that gives cosmetics a characteristic color, but also can act as bioactive ingredients. Moreover, the results of the analyses performed showed no cytotoxicity and a high antioxidant potential, which indicates no negative effect on the cells of various layers of the skin. The antioxidant and cytoprotective properties of the extracts depend on the concentration tested, but the highest cell proliferation capacity is shown by extracts from P. rhoeas L. and P. granatum L. The ability to scavenge free radicals and inhibit enzymes that degrade elastin and collagen fibers indicates the possibility of their use as ingredients in cosmetic and dermatological preparations intended to combat the aging process of the skin. In this case, the most favorable anti-aging properties are extracts from P. granatum L., but also G. globasa L. In addition, the extracts tested in this study provide long-term skin hydration, effectively protect against excessive water loss, and are characterized by a high SPF factor, thanks to which we can perceive them as effective natural UV filters (the most favorable for the extracts from P. granatum L. and C. tinctorius L.). The application analyses carried out showed that ***P. rhoeas* L.** (PRE), ***C. ternatea* L.** (KTE) and ***C. tinctorius* L.** (CTE) extracts may be used as effective colorants in cosmetics products. The creams containing them were marked by an intensive and stable color over time. Color of the cosmetics with the ***P. granatum* L.** (PGE) and ***G. globasa* L.** (GGE) extracts in the same concentration can be noticed only by experienced observers and they differ not significantly from base cream. These extracts did not turn out to be effective colorants for cosmetics creams. Due to the fact that many dyes used so far in cosmetic preparations are characterized by a negative impact on the skin and the natural environment, the results obtained in this study may become an impulse for manufacturers to consider the possibility of using these extracts as sustainable coloring ingredients with extraordinary biological properties.

## Figures and Tables

**Table 1:** Bioactive compounds detected using HPLC-ESI-MS in PRE, KTE, GGE, PGE and CTE extracts.

Retention Time (min)	Molecular Formula	Molar Mass (Da)	Precursorion *m*/*z*	Main Productions MS^2^ *m*/*z*	Identification	PRE	KTE	GGE	PGE	CTE
1.6	C_7_H_12_O_6_	192.2	191 [M − H]^−^	127 [M-H-H_2_O-HCOOH]^−^, 93 [M-C_4_H_3_O_3_]^−^, 85 [M-C_3_H_7_O_4_]^−^, 59 [M-C_5_H_9_O_4_]^−^	**Quinic acid**	x	x	x	x	x
2.1	C_7_H_6_O_5_	170.1	169 [M − H]^−^	151 [M-H-H_2_O]^−^, 125 [M-H-CO_2_]^−^, 107 [M-H-CO_2_-H_2_O]^−^,79 [M-C_6_H_3_O]^−^	**Gallic acid**	x	x	x	x	x
2.8	C_16_H_18_O_9_	354.3	353 [M − H]^−^	191 [M-3H_2_O-C_6_H_5_O_2_]^−^, 179 [M-3H_2_O-C_6_H_4_-COOH]^−^, 85 [M-C_15_H_12_O_2_-COOH]^−^,	**5-Caffeoylquinic acid**	x	x	-	x	x
4.2	C_16_H_18_O_9_	354.3	353 [M − H]^−^	191 [M-3H_2_O-C_6_H_5_O_2_]^−^, 179 [M-3H_2_O-C_6_H_4_-COOH]^−^, 85 [M-C_15_H_12_O_2_-COOH]^−^,	**3-Caffeoylquinic acid**	x	x	**<LOQ**	x	x
5.1	C_9_H_8_O_4_	180.2	179 [M − H]^−^	135 [M-COOH]^−^, 107 [M-C_3_H_5_O_2_]^−^,	**Caffeic acid**	x	x	x	x	x
7.1/8.1	C_27_H_30_O_16_	610.5	609 [M − H]^−^	300 [M-H-C_12_H_21_O_9_]^−^,	**Rutin**	x	x	x	x	x
8.5	C_27_H_30_O_15_	594.5	593 [M − H]^−^	383 [M-C_8_H_19_O_6_]^−^, 352 [M-C_9_H_21_O_7_]^−^, 284 [M-C_12_H_22_O_9_]^−^	**Kaempferol-3-*O*-rutinoside**	x	x	x	x	x
10	C_15_H_10_O_7_	302.2	301 [M − H]^−^	179 [M-H-C_7_H_6_O_2_]^−^, 151 [M-C_8_H_7_O_3_]^−^, 121 [M-C_8_H_5_O_5_]^−^,107 [M-C_9_H_5_O_5_]^−^	**Quercetin**	x	x	**<LOQ**	**<LOQ**	x
11.3	C_21_H_20_O_11_	448.3	447 [M − H]^−^	284 [M-H_2_O-C_6_H_10_O_4_]^−^,255 [M-C_6_H_9_O_7_]^−^, 179 [M-C_15_H_9_O_5_]^−^	**Kaempferol-3-*O*-glucoside**	x	x	x	x	x

**Table 2:** Quantification results obtained for PRE, KTE, GGE, PGE and CTE extracts. Values are means ± SD of triplicate experiments. Different letters indicate significant differences between groups (*p* < 0).

Compound	Content (µg/mL)				
	PRE	KTE	GGE	PGE	CTE
Quinic acid	0.14 ± 0.00 ^b^	2.63 ± 0.21 ^d^	0.05 ± 0.00 ^a^	0.87 ± 0.03 ^c^	5.95 ± 0.24 ^e^
Gallic acid	0.20 ± 0.02 ^b^	1.02 ± 0.01 ^c^	0.02 ± 0.00 ^a^	13.61 ± 0.13^d^	0.98 ± 0.01 ^c^
Caffeic acid	1.21 ± 0.03 ^c^	0.87 ± 0.01 ^b^	0.06 ± 0.00 ^a^	0.05 ± 0.00 ^a^	3.40 ± 0.03 ^d^
5-CQA	0.10 ± 0.00 ^b^	1.06 ± 0.05 ^c^	-	0.01 ± 0.00 ^a^	0.01 ± 0.00 ^a^
3-CQA	0.05 ± 0.00 ^a^	4.44 ± 0.00 ^d^	<LOQ	0.58 ± 0.00 ^b^	0.67 ± 0.00 ^c^
Quercetin	16.05 ± 0.27 ^c^	5.08 ± 0.00 ^b^	<LOQ	<LOQ	0.86 ± 0.01 ^a^
**Sum of quantified compounds**	**17.75**	**15.1**	**0.13**	**15.12**	**11.87**

**Table 3:** Color parameters for water–ethanol extracts (concentration of 100 mg/mL): *P. rhoeas* L. (PRE), *P. granatum* L. (PGE)*, C. ternatea* L. (KTE), *C. tinctorius* L. (CTE)*, G. globasa* L. (GGE). Values are means of three replicate determinations (*n* = 3) ± SD.

	L*	a*	b*	C*	h^o^	
**PRE**	14.19±0.09	3.04 ± 0.03	2.02 ± 0.01	3.7 ± 0.03	33.4 ± 0.03	**red**
**PGE**	22.48 ± 0.10	−0.56 ± 0.01	2.42 ± 0.05	2.5 ± 0.05	102.9 ± 0.04	**yellow**
**KTE**	13.59 ± 0.11	2.05 ± 0.02	−0.93 ± 0.01	2.2 ± 0.02	335.6 ± 0.03	**Blue–violet**
**CTE**	18.95 ± 0.07	0.17 ± 0.05	8.22 ± 0.01	8.2 ± 0.05	88.8 ± 0.03	**yellow**
**GGE**	22.39 ± 0.09	−0.65 ± 0.02	2.38 ± 0.03	2.5 ± 0.04	105.4 ± 0.04	**yellow**

**Table 4:** Color parameters for cosmetic creams with water–ethanol extracts: *P. rhoeas* L. (PRE), *P. granatum* L. (PGE)*, C. ternatea* L. (KTE), *C. tinctorius* L. (CTE)*, G. globasa* L. (GGE). Values are means of three replicate determinations (*n* = 3) ± SD.

	L*	a*	b*	C*	h^o^	ΔE cream+Extract/Base Cream	
**Cream with PRE**	81.48 ± 0.09	5.34 ± 0.03	2.81 ± 0.01	6.0 ± 0.03	27.8 ± 0.1	11.88 ± 0.02	**red**
**Cream with PGE**	90.83 ± 0.10	−0.84 ± 0.01	2.94 ± 0.05	3.1 ± 0.05	105.9 ± 005	1.63 ± 0.04	**white, slightly yellow shade**
**Cream with KTE**	68.19 ± 0.11	6.23 ± 0.02	18.92 ± 0.01	19.9 ± 0.02	288.2 ± 0.02	31.71 ± 0.04	**Blue–violet**
**Cream with CTE**	88.71 ± 0.07	−5.95 ± 0.05	23.46 ± 0.01	24.2 ± 0.05	104.2 ± 0.04	22.72 ± 0.03	**yellow**
**Cream with GGE**	91.20 ± 0.09	−0.88 ± 0.02	3.00 ± 0.03	3.1 ± 0.04	106.4 ± 0.04	1.59 ± 0.02	**white, slightly yellow shade**
**Base cream**	91.40 ± 0.07	−1.06 ± 0.02	1.43 ± 0.02	1.8 ± 0.03	306.6 ± 0.04	-	**white, slightly purple shade**

**Table 5:** Formulation of a model cream for facial skin care.

	Ingredient (INCI Name)	Raw Material (Trade Name, Supplier)	(wt.%)
**1**	Helianthus Annuus (Sunflower) Seed Oil	Sunflower Oil (local supplier)	8.0
**2**	Butyrospermuum Parkii (Shea) Butter	Cetiol SB 45 (BASF)	6.0
**3**	Coco-Caprylate/Caprate	Cetiol LC (BASF)	2.0
**4**	Glyceryl Stearate	Cutina GMS V (BASF)	1.0
**5**	Polyglyceryl-3 Dicitrate/Stearate	TegoCare PSC-3 (Evonik)	2.0
**6**	Cetearyl Alcohol	Lanette O (BASF)	4.0
**7**	Glycerin	Glycerin Vege.7 (local supplier)	2.0
**8**	Propanediol	Propanediol natural (Cosphaderm)	2.0
**9**	Aqua	Deionised water	to 100
**10**	Benzyl Alcohol, Benzoic Acid, Dehydroacetic Acid, Tocopherol	Euxyl K903 (Schülke & Mayr)	0.5
**11**	Sodium Hydroxide/ Lactic Acid	NaOH/Lactic Acid (local supplier)	to pH 5.5

## Data Availability

Data is contained within the article.

## Supplementary Materials

Supplementary materials (chromatograms) are available online.

## Sample Availability

Samples of the compounds used and the plants extracts tested are available from the authors.
