# Supplementary figures and images for: Antioxidant and Cytoprotective Properties of Plant Extract from Dry Flowers as Functional Dyes for Cosmetic Products
Source: Molecules. 2021 May 10;26(9):2809. doi: 10.3390/molecules26092809 (PMC8126054; doi:10.3390/molecules26092809)

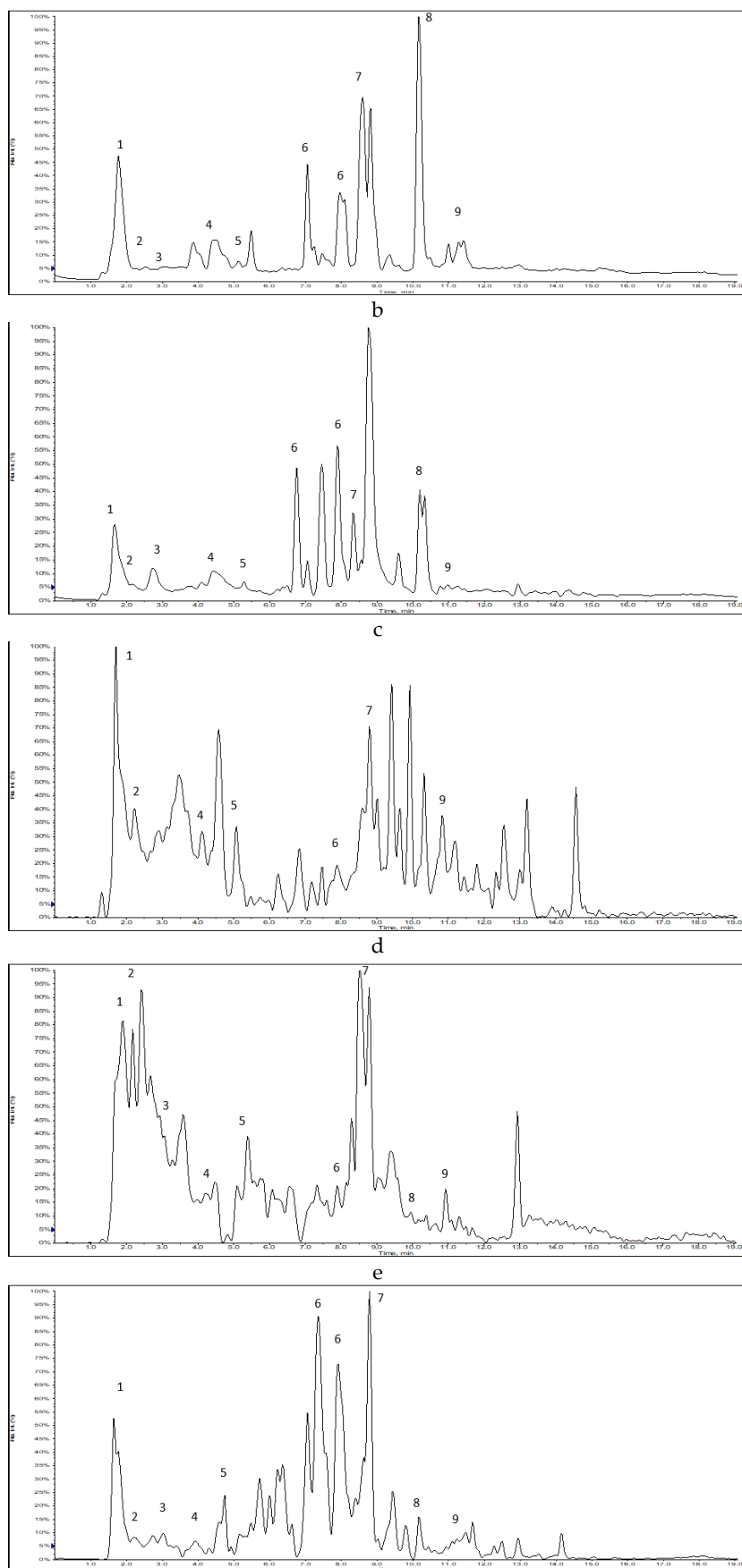

**Figure S1.** Extracted ion chromatograms (XIC) obtained for a) PRE, b) KTE, c) GGE, d) PGE and e) CTE.

Supplement: Supplementary file 1 [file molecules-26-02809-s001.zip › molecules-1204918-supplementary.pdf]
